# Antioxidant status in relation to heavy metals induced oxidative stress in patients with polycystic ovarian syndrome (PCOS)

**DOI:** 10.1038/s41598-021-02120-6

**Published:** 2021-11-25

**Authors:** Manal Abudawood, Hajera Tabassum, Atheer H. Alanazi, Fatmah Almusallam, Feda Aljaser, Mir Naiman Ali, Naif D. Alenzi, Samyah T. Alanazi, Manal A. Alghamdi, Ghadah H. Altoum, Manar A. Alzeer, Majed O. Alotaibi, Arwa Abudawood, Hazem K. Ghneim, Lulu Abdullah Ali Al-Nuaim

**Affiliations:** 1 Chair of Medical and Molecular Genetics Research, Department of Clinical Laboratory Sciences, College of Applied Medical Sciences, King Saud University, Riyadh, Saudi Arabia; 2 Department of Clinical Laboratory Sciences, College of Applied Medical Sciences, King Saud University, Riyadh, Saudi Arabia; 3 Microbiology Section, Riyadh Municipality Central Area Labs, Riyadh, Saudi Arabia; 4 Research and Laboratories Sector, National Drug and Cosmetic Control Laboratories (NDCCL), Saudi Food and Drug Authority, Riyadh, Saudi Arabia; 5 Pharmaceutical Quality Control and Quality Assurance Program, Riyadh Elm University, Riyadh, Saudi Arabia; 6 Department of Family and Community Medicine, Prince Sultan Military Medical City, Riyadh, Saudi Arabia; 7 Reproductive Medicine and Endocrinology, College of Medicine, King Saud University, Riyadh, Saudi Arabia

**Keywords:** Biochemistry, Biomarkers, Diseases, Medical research, Risk factors

## Abstract

Polycystic ovary syndrome (PCOS) is a global health concern for women of reproductive age, as 6.5% of women worldwide are affected by this syndrome. PCOS is marked by hyperandrogenism, anovulation, menstrual abnormalities, and polycystic ovaries. Metals such as arsenic, cadmium, lead and mercury are considered to be systemic toxicants/human carcinogens and seem to have devastating effects on humans, even at minimal exposures. One of the probable aetiological factors for PCOS has been identified as oxidative stress. In view of the probable associations among oxidative stress, metal toxicity and PCOS, the present study examined the role of heavy metals in the generation of oxidative stress among females. This prospective study included 106 women (56 women diagnosed with PCOS and 50 women who were not diagnosed with PCOS as control women). There were no significant differences in the sociodemographic characteristics between the two groups except for the irregularity of menses and the presence of acne. The serum As, Cd, Pb, and Hg levels increased and the serum glutathione (GSH) and superoxide dismutase (SOD) levels diminished significantly in the PCOS group compared to the control group at *P* < 0.001. The SOD levels were negatively correlated with the As and Pb levels at *P* < 0.05. Additionally, the PCOS group exhibited a strong negative correlation between the GSH and As levels (*P* < 0.01), GSH and Pb levels (*P* < 0.05) and GSH and Hg levels (*P* < 0.01). Furthermore, the As levels were positively correlated with increased levels of Cd, Pb and Hg among PCOS women. Significant positive correlations were observed between Pb and Cd and between Cd and Hg at *P* < 0.001. The outcome of the study provides clear insight into the role of metal-induced oxidative stress, which plays a vital role in the pathophysiology underlying PCOS and suggests the use of these markers as prognostic tools to reduce the consequences of high-risk exposure to these metals among females.

## Introduction

Approximately 10% of women are affected by infertility, among which 6.5–8% of women of reproductive age are affected by polycystic ovary syndrome (PCOS), which constitutes the most prevalent cause of infertility among females^1^. As a type of endocrinopathy, PCOS is characterized by chronic anovulation, menstrual abnormalities (e.g., oligomenorrhea or amenorrhea), hyperandrogenism, hyperinsulinaemia and polycystic ovaries^2,3^. However, the exact aetiology that underlies PCOS is still unresolved. Among the naturally existing metals which are ubiquitous in nature, some metals possess high specific density > 5 g/cm^3^ and atomic weight > 40 and are termed as ‘heavy metals’ like cadmium (Cd) lead (Pb), arsenic (As) etc. These heavy metals have deleterious effects resulting in acute and chronic toxicities in living organisms. Whilst, other heavy metal like zinc, iron, and copper are required for normal physiological and biochemical functioning of the body. Nonetheless, some of the heavy metals in this category are harmful at high doses^4^.The environment is constantly being polluted by heavy metals from industries, and exposure to these metals is a major area of public health concern, especially for women of childbearing age, as it can cause reproductive dysfunction in women^5,6^. The main routes of exposure in the environment are soil, air, polluted water, smoking, and food^7^. Quite a few studies have demonstrated the antagonistic effects of heavy metals in utero^8,9^. Heavy metals may induce hormonal changes that affect the menstrual cycle, ovulation, and female fertility^10^. Nonessential metals such as lead (Pb), cadmium (Cd), and arsenic (As) are reproductive toxicants that are widely distributed in the environment and require close monitoring^11^. The role of heavy metals in altering hormonal levels has been evidenced by several epidemiologic studies^12,13^. However, studies that have investigated the impact of heavy metals on the aetiology of PCOS are scarce. Women with blood Pb levels higher than 25 μg/L were reported to have a threefold increased risk of infertility compared to women with Pb levels less than 25 μg/L^14^. Similarly, Cd was reported to influence fertility hormones; for every 1 μg/L increase in Cd levels, a 21% increase in the levels of early follicular phase oestradiol (E2); serum follicle-stimulating hormone (FSH) and luteinizing hormone (LH) concentrations was observed^15^.

In this report, we assume that heavy metal intoxication could lead to oxidative stress (OS) and is responsible for the pathophysiology that underlies this disease. Metals such as As^16^, Cd^17^, Cr^18^, Pb^19^, and Hg^20^ are among the most toxic elements and are considered to be systemic toxicants due to multiple organ damage (even at minimal exposures). Metal ions can trigger ROS production and/or have antagonistic actions on the antioxidant status of cells, which can lead to OS. Heavy metals can inevitably cause conformational changes in DNA and/or other nuclear proteins by binding to them and altering the events of the cell cycle, which might lead to apoptosis/cancer^21^. In short, several lines of evidence suggest that metals might be involved in the development of PCOS. However, previous studies in developed settings have documented inconsistent findings among PCOS patients in terms of activities of antioxidant status, and some have reported no significant differences among PCOS females. Hence, more studies are necessary to further investigate the relationships involving antioxidant status and PCOS. In view of the existence of a correlation between oxidative stress and PCOS, the present study hypothesizes the role of heavy metal toxicity in the generation of oxidative stress in PCOS. From the above perspectives, the current study aimed (a) to determine the serum concentrations of heavy metals and antioxidant markers in PCOS patients and the controls and (b) to investigate the correlations among heavy metal concentrations and antioxidant markers in PCOS patients.

## Result

The current report evaluated the sociodemographic data, oxidative stress biomarkers, and heavy metal (e.g., As, Cd, Pb and Hg) levels between the PCOS and control groups. Most of the participants were young and married. Their sociodemographic characteristics (outlined in Table 1) were less likely to confound the obtained results. For the 106 women, 47.1% were controls, and 52.8% were women with PCOS. The majority of the women were married and were in the age group of 19 to 35 years. There were no statistical differences in the sociodemographic features that were studied in these groups. However, a proportion of the women who demonstrated irregular menses (56%) and acne (60%) exhibited significant differences in these characteristics. A comparison of their biochemical characteristics together with their antioxidant status is shown in Table 2. The serum fasting blood sugar (FBS) and HbA1c levels were found to increase in the PCOS group compared to the control group. These elevated levels varied significantly at *P* < 0.001. Elevated levels of luteinizing hormone (LH) and triglycerides (TGs) were found to be significant in the PCOS group at *P* < 0.001 and *P* < 0.05, respectively. The mean serum SOD values decreased significantly in the PCOS group (9.30 ± 3.2 IU/ml) compared to the control (17.39 ± 3.35 IU/ml). In parallel, a significant drop in the serum GSH levels was observed in the PCOS group (6.24 ± 1.50 mg/ml) compared to the control group (8.09 ± 1.39 mg/ml). The decreased antioxidant status that was observed in the PCOS patients was significant at *P* < 0.001 (Table 2).

**Table 1:** Sociodemographic and clinical characteristics of the study groups.

	Control	PCOS	*P*
	n (%)	n (%)	
Age (mean ± SD)	29.16 ± 6.2	30.41 ± 6.8	NS
Marital status			NS
Married	30 (53)	24 (48)	
Unmarried	26 (46.4)	26 (52)	
Irregular menses	4 (7.1)	28 (56)	0.002**
Acne	27 (48.2)	30 (60)	0.04*
Skin pigmentation	16 (28.5)	23 (46)	NS
Cases of nipple discharge	4 (7.1)	NIL	NS
Oral contraceptives	2 (3.5)	2 (4)	NS
History of hypertension	1 (1.78)	2 (4)	NS
Gestational diabetes (GD)	1 (1.78)	NIL	NS
Cardiovascular diseases (CVDs)	1 (1.78)	NIL	NS

NS, non-significant.

*p ≤ 0.05, **p ≤ 0.01.

**Table 2:** Biochemical characteristics and antioxidant status of the study groups.

	Control	PCOS	*P*
Age	29.16 ± 6.2	30.41 ± 6.8	NS
BMI	25.0 ± 6.08	27.23 ± 5.0	NS
FBS (mmol/L)	5.2 ± 0.9	6.88 ± 1.49	**<**0.001
HbA1c (%)	5.37 ± 0.30	6.70 ± 0.22	**<** 0.001
TC (mmol/L)	4.48 ± 0.96	4.36 ± 0.9	0.57
TG (mmol/L)	1.37 ± 0.77	1.89 ± 0.32	0.02*
HDL (mmol/L)	1.31 ± 0.35	1.24 ± 0.34	0.32
LDL (mmol/L)	2.50 ± 0.84	2.79 ± 0.98	0.09
LH (mmol/L)	2.95 ± 0.75	6.73 ± 0.1	**<** 0.001
SOD (IU/ml)	17.39 ± 3.35	9.30 ± 3.2	**<** 0.001
GSH (mg/ml)	8.09 ± 1.39	6.24 ± 1.50	**<** 0.001

NS, non-significant.

*p ≤ 0.05.

**Figure 1:**
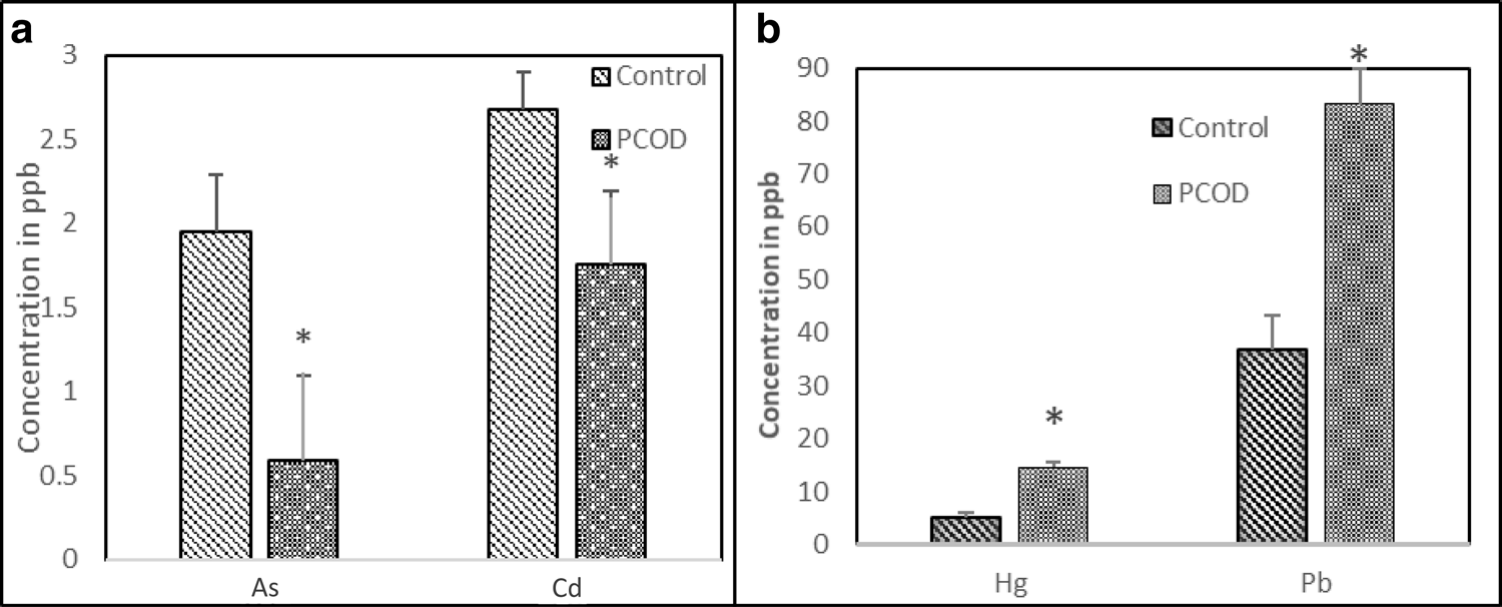
Serum concentrations of heavy metals in control and PCOS groups.

In contrast to the investigated antioxidant markers, the PCOS patients exhibited marked increases in their heavy metal levels compared to the controls. The mean serum values of As, Cd, Pb and Hg were 1.95 ± 0.34, 0.59 ± 0.22, 36.69 ± 6.57 and 5.0 ± 1.08 ppb in the control group and 2.68 ± 0.50, 1.75 ± 0.44, 83.19 ± 14.4 and 14.55 ± 2.99 ppb in the PCOS group, respectively. Figure 1 a,b show the variations in the of As, Cd, Pb and Hg levels between the two groups, with a significant increase in heavy metals in the PCOS group compared to the control (*P* < 0.001). The correlations among heavy metals, antioxidants and other metabolic markers were determined from the Pearson correlation (*r*) values, as shown in Table 3. The interelement relationships of the investigated heavy metals are depicted in Table 4. Most of the studied elements exhibited nonsignificant correlations with the studied metabolic markers, BMI, FBS and lipid profile parameters, with the exception of Hg, which exhibited a positive significant correlation with FBS and HbA1c at (*P* < 0.001 and *P* < 0.05, respectively). Nevertheless, Cd was positively correlated with total cholesterol (TC) (r = 0.30, *P* < 0.05). A strong significant negative correlation between increased HbA1c and decreased antioxidant (SOD) at *P* < 0.001 was also obtained. Additionally, the SOD was significantly correlated with decreased LH at *P* < 0.05.

**Table 3:** Correlation coefficients among the oxidative stress markers, metabolic markers and heavy metals in the PCOS group.

	As r(*P*)	Cd r(*P*)	Pb r(*P*)	Hg r(*P*)	SOD r(*P*)	GSH r(*P*)
BMI	0.06 (0.73)	0.06 (0.74)	0.23 (0.20)	0.29 (0.11)	0.04 (0.8)	0.17 (0.30)
FBS	0.09 (0.50)	− 0.02 (0.88)	− 0.03 (0.78)	0.29 (0.004)**	− 0.26 (0.06)	− 0.19 (0.18)
HbA1c	0.01 (0.90)	0.22 (0.12)	− 0.18 (0.18)	0.27 (0.05)*	**−** 0.32 (0.02)*	0.006 (0.99)
TC	− 0.09 (0.49)	0.30 (0.02)*	− 0.05 (0.68)	0.14 (0.30)	0.11 (0.43)	0.02 (0.84)
TG	0.02 (0.84)	− 0.07 (0.62)	0.02 (0.85)	0.01 (0.30)	0.13 (0.33)	− 0.19 (0.17)
HDL	− 0.02 (0.86)	− 0.19 (0.18)	− 0.07 (0.61)	− 0.03 (0.80)	− 0.04 (0.77)	0.14 (0.32)
LDL	0.007 (0.96)	− 0.18 (0.20)	− 0.01 (0.96)	0.15 (0.2)	0.10 (0.48)	0.004 (0.97)
LH	0.003 (0.84)	− 0.19 (0.17)	− 0.16 (0.25)		0.03 (0.007)**	− 0.02 (0.86)
SOD	**−** 0.1 (0.04)**	− 0.11 (0.40)	**−** 0.32 (0.02)*	− 0.16 (0.21)	–	− 0.04 (0.73)
GSH	**−** 0.41 (0.002)**	− 0.16 (0.22)	**−** 0.24 (0.04)*	**−** 0.023 (0.0098)*	− 0.04 (0.73)	–

*p ≤ 0.05; **p ≤ 0.01.

**Table 4:** Interelement relationships of heavy metals in PCOS cases. *p ≤ 0.05

	As r(*p*)	Cd r(*p*)	Pb r(*p*)
Cd	0.54 **(*p*** **<** **0.001)**	–	0.29 (0.02)*
Pb	0.54 **(*p*** **<** **0.001)**	0.29 (0.02)*	–
Hg	0.47 **(*p*** **<** **0.001)**	0.52 **(*p*** **<** **0.001**)	0.44 (***p*** **<** **0.001)**

**Figure 2:**
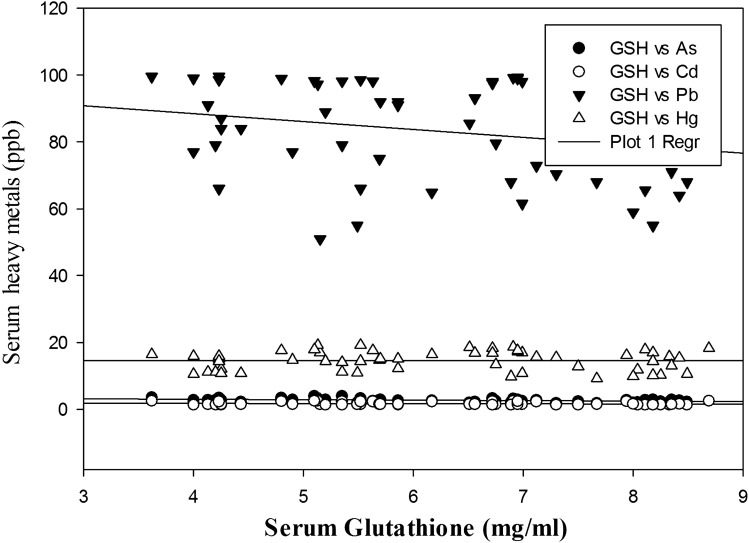
Multiple regression plot of heavy metals and GSH in PCOS group.

**Figure 3:**
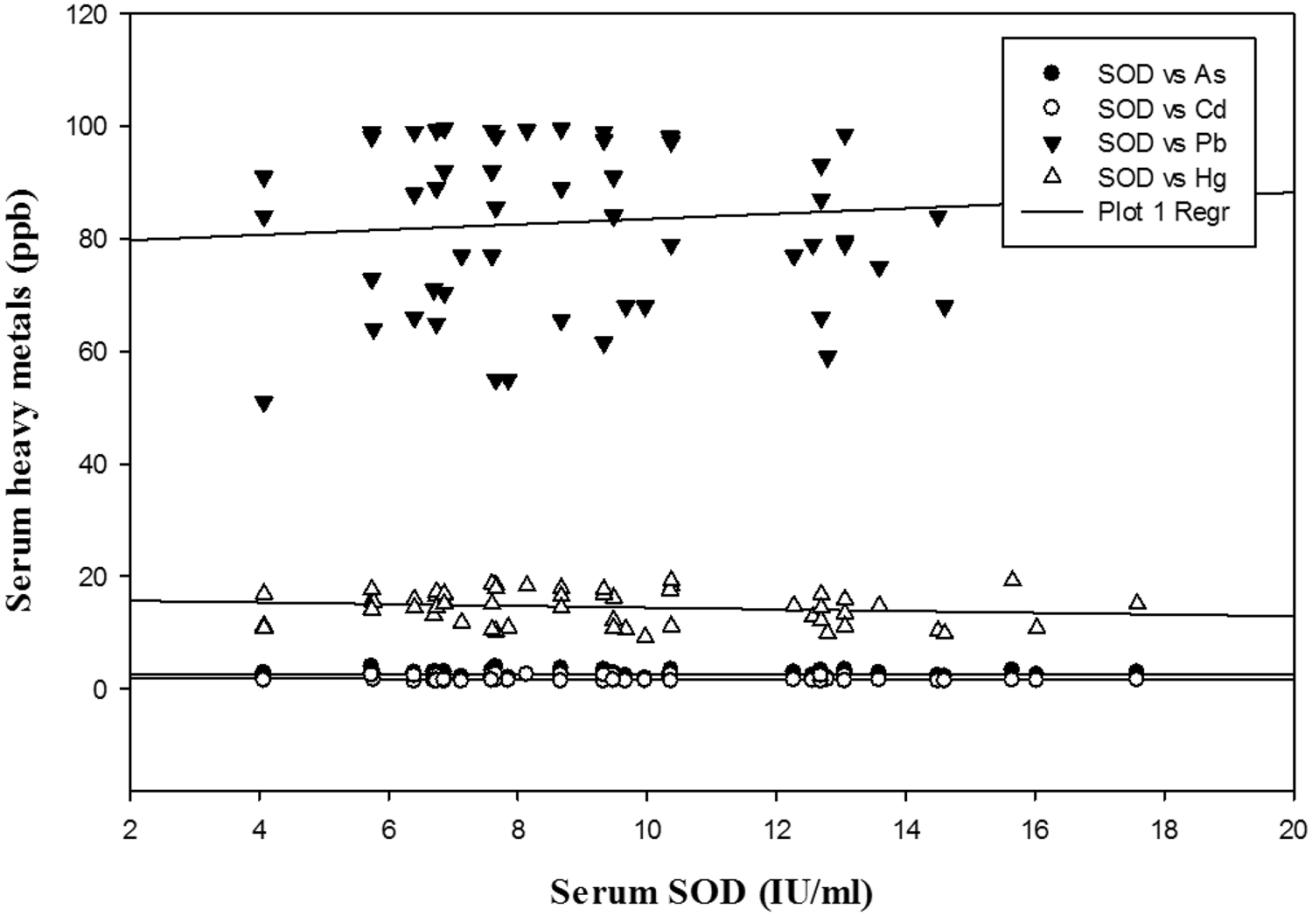
Multiple regression plot of heavy metals and SOD in PCOS group.

Intriguingly, the Pearson correlation that was performed to evaluate the impact of heavy metals on oxidative stress markers yielded satisfactory results. As, Pb and Hg exhibited strong negative correlations with GSH at *P* < 0.01, *P* < 0.05, and *P* < 0.01 respectively. Furthermore, SOD was negatively correlated with As and Pb (*P* < 0.05) among women with PCOS. Moreover, strong positive correlations were observed among As and other metals (e.g., Cd, Pb and Hg), which were statistically significant at *P* < 0.001.The correlations between Cd and Pb and between Cd and Hg were statistically significant at *P* < 0.05 and *P* < 0.001, respectively. The serum levels of Hg and Pb also exhibited a strong positive correlation, which was statistically significant at *P* < 0.001. The correlations among the heavy metals and antioxidant markers are represented in the form of multiple regression plots (Figs. 2 and 3).

## Discussion

The main findings of the current study were the elevated levels of heavy metals with a diminished antioxidant status for the PCOS women compared to the non-PCOS women.The impact of heavy metals on oxidative stress, which constitutes a paramount cause in the aetiology of PCOS, was evidenced here. There were no significant differences in the sociodemographic characteristics between the studied groups, yet there were some significant variations in certain variables, such as irregular menses and acne problems. The serum GSH and SOD levels decreased significantly between the two groups (*P* < 0.001). There were strong negative correlations between GSH and As (*P* < 0.01), GSH and Pb (*P* < 0.05) and GSH and Hg (*P* < 0.01). SOD was negatively correlated with As and Pb at *P* < 0.05. Furthermore, significant positive correlations was observed between Pb and Cd and between Cd and Hg at *P* < 0.001.

Inspite of the extensive research on PCOS, its aetiology is still obscure. PCOS is intricated with a cascade of impaired metabolism. Abnormalities in lipids and lipoproteins (dyslipidaemia) could be viewed as possible complications of PCOS, as evidenced by the higher TG levels in women with PCOS than in the controls. The lack of a significant association between the lipid parameters and heavy metals reflects the role of the lipid profile as an independent variable in the aetiology of PCOS. In addition, the LH hormone was negatively correlated with decreased SOD, which indicated that the levels of fertility hormones are influenced by oxidative stress in the human body, as is evident in the current report.

As evidenced in the current report, the decreased SOD activity and GSH in women with PCOS could be due to a surplus production of free radicals that is produced by metal intoxication. In parallel with the current observations, Hilali et al. (2013) reported a diminished antioxidant status among the PCOS vs. control groups^22^. The decreased GSH among women with PCOS, as observed in the current report, are in accordance with previous findings^23,24^. Interestingly, a similar trend in antioxidant status was observed with respect to SOD. Diminished SOD levels that were observed in the current study in women with PCOS are homologous to previous findings^25,26^ but not with certain other works^27^ that demonstrated significantly higher SOD activities in PCOS patients. In contrast, Yilmaz et al. (2016) reported no significant changes between the test and control groups^28^. This inconsistency in the data might have been due to a compensatory response by the body’s defence mechanisms to the higher circulating levels of oxidants. Heavy metal elements can act as endocrine disruptor chemicals (EDCs) by generating OS^29,30^. Disturbances in the normal oxidation–reduction reactions of cells with the overproduction of free radicals or reactive oxygen species (ROS) and peroxides result in OS that can cause toxic effects/cell damage. Interestingly, cells harbour molecules called antioxidants that detoxify ROS and prevent OS. Antioxidants include highly complex enzymatic and nonenzymatic molecules/systems. GSH is a nonenzymatic antioxidant, and SOD is an enzymatic antioxidant, and these two substances constitute prominent antioxidant markers that indirectly predict the OS status in cells. GSH functions as an important antioxidant that performs vital cellular functions^31^. GSH catalyses the detoxification of oxidizing compounds via its thiol groups^32^. SOD functions by catalysing the detoxification of superoxide anions (O_2_^−^), as major oxygen radicals, to H_2_O_2_ and finally to water^33^. SOD exists in different forms with Cu/Zn, Fe and Mn as cofactors^34^.

There are numerous sources by which humans are exposed to heavy metals, such as occupational exposure, environmental pollution, and/or food consumption. Heavy metals, including Cd, Pb, and As, are ubiquitous in the environment due to the many years of industrial use, and most adults have measurable levels of these nonessential elements in their blood. The results from previous reports on the levels of trace elements in women with PCOS are conflicting. In a study by Zheng et al. (2015), no significant changes were reported for the levels of these heavy metals in Chinese women^35^. In contrast, Kirmizi et al. (2020) demonstrated increased levels of Cd, Hg and Pb between the two groups^23^. Intriguingly, data of the heavy metals (e.g., As, Cd, Pb and Hg) that were investigated in the present study are in accordance with previous findings^23^. Nevertheless, with respect to As, the levels exhibited a significant difference in contrast to the finding of Kirmizi et al. (2020)^23^. Leafy vegetables, grains, crustaceans, mushrooms, shellfish, mussels, liver and kidney are a few of the food sources that contain significant amounts of Cd and are responsible for Cd intoxication^36^. Cd intoxication causes deleterious effects on cellular functioning by indirectly synthesizing ROS. Presumably, Cd-induced OS includes alterations in thiol proteins, metabolic and endocrine inhibition, and alterations of metalloenzymes, DNA and other vital molecules^37^. The reproductive and teratogenic effects of Cd have also been studied in animal models. Numerous studies have investigated the relationship of cadmium with female reproductive disorders. Elevated Cd levels in women with PCOS are inconsistent with the finding of Kurdoglu et al. (2012). In a Turkish-based study on PCOS females, Kurdoglu et al. (2012) reported no significant differences in the Cd levels and reported decreased Pb levels among these females^38^. Nevertheless, the increased Cd levels did not exhibit any significant associations with antioxidant markers, as was observed in previous findings^23^. As is detrimental to the human body and affects various organ and organ systems^39^. In addition to Cd and As, another important, widespread environmental toxicant/pollutant is Hg. It has been determined to cause adverse health effects by inducing rigorous alterations in the human body^40^. The various routes of exposure to Hg can occur through occupational operations, environmental pollution, dental care, preventive medical practices, and industrial and agricultural operations. Notably, dental amalgams and fish are recognized as chronic sources of Hg^41^. The effects of chronic and relatively low Hg exposure are known to inhibit enzymatic activity, which can thereby induce OS, and can sometimes be genotoxic to cells^42^. Occupational and experimental Hg exposures have shown that Hg induces several reproductive and metabolic abnormalities, such as reproductive cyclicity disturbances, irregular ovarian follicular development, ovulation inhibition, infertility, spontaneous miscarriage, increased visceral adiposity, risk of diabetes mellitus, and metabolic syndrome in rodent and human models^40,43^. In addition to As and Cd, Pb and Hg are also known to exert deleterious effects on human health by cellular dysfunction, which generates OS. The increased Hg levels among PCOS patients are in line with previous findings^23^.

Furthermore, the Pearson correlation analysis revealed significant negative associations among the antioxidant markers and concentrations of the investigated heavy metals (Table 3). Figures 2 and 3 depict the multiple regression analysis between heavy metals and antioxidant markers. A negative correlation between Pb and two antioxidant markers (SOD and GSH) is reflective of the decreased antioxidant status among PCOS females due to oxidation of glutathione, which causes a reduction in serum GSH levels^23^. Nonetheless, elevated serum Hg levels in PCOS females were negatively correlated with GSH levels and indicated the oxidative property of Hg in the generation of OS. By further exploring the interelement relationships among the heavy metals, the present study demonstrated significant positive correlations among the heavy metals (e.g., As, Cd, Pb and Hg), which indicated that the pathophysiology that develops in PCOS is probably due to the enhanced levels of these heavy metals that work in concert and lead to OS.

The strength of the study, is its assessment of heavy metals in association with oxidative stress as pathophysiological process underlying PCOS. However, the current study has some limitations. The study does not evaluate the potential exposure levels of toxic metals from the residential areas of the participants; that might affect the metal levels in blood. Further, larger studies involving a large sample size are needed for clear elucidation of the role of heavy metals in PCOS in relation to OS.

## Conclusion

The results obtained in the present study are indicative of the role of heavy metal-induced oxidative stress as one of the vital aetiological factors involved in the pathogenesis of PCOS. Oxidative stress and heavy metal toxicity should be regularly monitored in females to reduce the risk of developing PCOS. These tests must be integrated in the diagnoses of PCOS along with conventional biochemical parameters from the early stages to ensure a healthy status among females of reproductive age.

## Methodology

This case-controlled study consisted of two groups of women in the age group of 19 to 35 years: Group-I; control (56) and Group-II; PCOS patients (50). The patients were screened based on the Rotterdam criteria^11^ and were categorized as PCOS positive if they had at least two features: (a) oligo- or amenorrhea (< 8 menstrual cycles in the current year), (b) hyperandrogenism or (c) polycystic ovaries. Healthy females with no symptoms of hyperandrogenism, history of menstrual dysfunction, infertility, or sonographic signs of PCOS were treated as the controls. This study was carried out in the Department of Clinical Laboratory Sciences, King Saud University in collaboration with the Section of Obstetrics and Gynaecology, King Khalid University Hospital (KKUH), Riyadh, KSA, from October 2018 to December 2020. The Institutional Review Board, KKUH approved the study (E-18–3536). All methods were performed in accordance with the relevant guidelines and regulations from the ethical committee. Informed consent was obtained from patients. A trained interviewer administered a standard face-to-face questionnaire to each participant to obtain the potential factors that might reveal the body burden of metals, including sociodemographic information, lifestyle characteristics, anthropometry, and menstruation history. Pregnant women and women with diabetes mellitus and taking lipid-lowering or antihypertensive drugs, anaemia, malignant neoplasia, any active infectious diseases or thromboembolism, stroke or history of ischaemic heart disease were excluded from the study.

### Sample collection and preliminary investigations

Blood samples (5 ml) were drawn from each subject who participated in the study. The blood samples were subjected to centrifugation at 1500 ×*g* for 15 min to obtain the serum. The serum samples were transferred to eppendorf tubes and stored at − 80 °C until analysis. All preliminary investigations, including the CBC and lipid profiles, were analysed in an Auto analyser, Cell Dyne 3700 (STA compact, Mediserv, UK). The LH measurements were performed with a Roche Elecsys 2010 Modular Analytics E170-Cobas e 411 that utilized an electrochemiluminescence immunoassay (Roche Diagnostics, Germany).

### Determinations of superoxide dismutase activity

The SOD activities were measured by an SOD Assay Kit-WST (19160), Sigma. A WST working solution and enzyme working solution were added to blank and study samples in a 96-well plate. After an incubation time of 20 min at 37 °C, the absorbances were read at 450 nm using a plate reader. The SOD activities were calculated and expressed as IU/ml.

### Determinations of Glutathione content

Glutathione Assay Kit (CS0260) Sigma was used to estimate the serum glutathione levels. This kit mainly employs a kinetic assay in which catalytic amounts (nmoles) of GSH cause a continuous reduction of 5,5′-dithiobis(2-nitrobenzoic acid) (DTNB) to TNB and the GSSG that forms is recycled by glutathione reductase and NADPH. The reaction rate is proportional to the glutathione concentration up to 2 µM/minute. The yellow product, 5-thio-2-nitrobenzoic acid (TNB), was measured for 5 min spectrophotometrically at 412 nm.

### Heavy metal analysis by inductively coupled plasma mass spectrophotometry (ICP-MS)

An inductively coupled plasma mass spectrophotometer (ICP/MS), Agilent Technologies 7700, was employed to assay the heavy metal levels in the serum samples. Prior to analysis, the serum samples (400 µl) were centrifuged and diluted with solvent mix (2.5 ml) consisting of 1% HNO3 and 0.01% Triton × 100 (HPLC grade, Sigma Aldrich). The determinations of all heavy metals were performed by running a calibration curve with a detection range of 0.05–100 ppb (prepared from a standard stock solution 1000 ppb).

### Statistical analyses

The statistical analyses was performed using SigmaPlot software. The sociodemographic data were analysed by the Wilcoxon signed rank test to ascertain the significant differences between the studied groups. The comparisons of the clinical characteristics, biochemical parameters and heavy metal levels were performed by the use of paired t tests. The Pearson correlation and multiple regression analyses were applied to the study parameters to investigate the role of heavy metals on the antioxidant status.
